# A loop cutter is an ideal gripper for endoscopic removal of press-through-package sheets

**DOI:** 10.1055/a-2113-9265

**Published:** 2023-07-13

**Authors:** Yuji Ino, Masafumi Kitamura, Yoshie Nomoto, Chihiro Iwashita, Yoshimasa Miura, Tomonori Yano, Hironori Yamamoto

**Affiliations:** Department of Medicine, Division of Gastroenterology, Jichi Medical University, Shimotsuke, Japan

A press-through package (PTP) sheet is sometimes accidentally swallowed 1 . PTP sheets have sharp edges ( Fig. 1 ) and may cause mucosal damage and/or perforation to the gastrointestinal tract 2 3 . Thus, ingested PTP sheets should be removed endoscopically as soon as possible. Several methods have been reported using hoods and covers to prevent mucosal damage during removal 4 5 . However, a firm grip on the PTP sheet is necessary for successful removal. Grasping forceps or a snare are commonly used as grasping devices but can often fail because the PTP is slippery with saliva and the working space is insufficient for snaring. To address this, we used a Loop Cutter (FS-5L-1; Olympus, Tokyo, Japan), whose teeth can penetrate the PTP plastic sheet to secure a firm grip. The Loop Cutter’s strong grip allows the PTP to be easily removed.

**Fig. 1:**
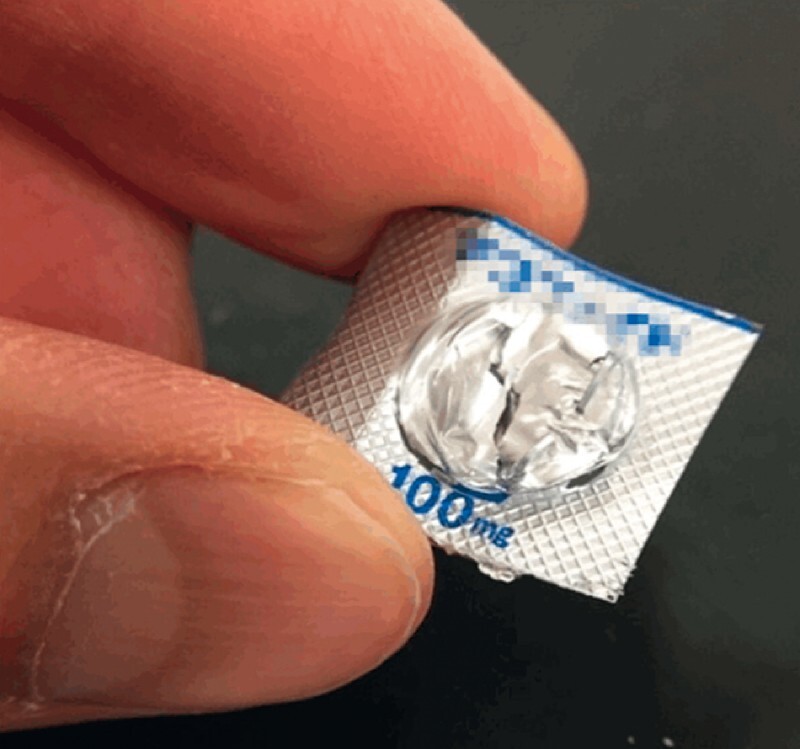
Press-through-package (PTP) sheets have sharp edges.

To evaluate the gripping force of various devices, we attached a PTP sheet to the tip of a spring scale ( Fig. 2 ). The gripping force was measured until the PTP was detached from the grasping device. The mean gripping force of each thirty times was 498 g in Standard Fenestrated Biopsy Forceps (FB-25K-1, Olympus), 404 g in the Radial Jaw 4 Pediatric Biopsy Forceps (M00513440; Boston Scientific, Marlborough, Massachusetts, USA), 37 g in the Rat Tooth with Alligator Jaw Grasping Forceps (FG-47L-1; Olympus), and 1440 g in the Loop Cutter ( Fig. 3 ).

**Fig. 2:**
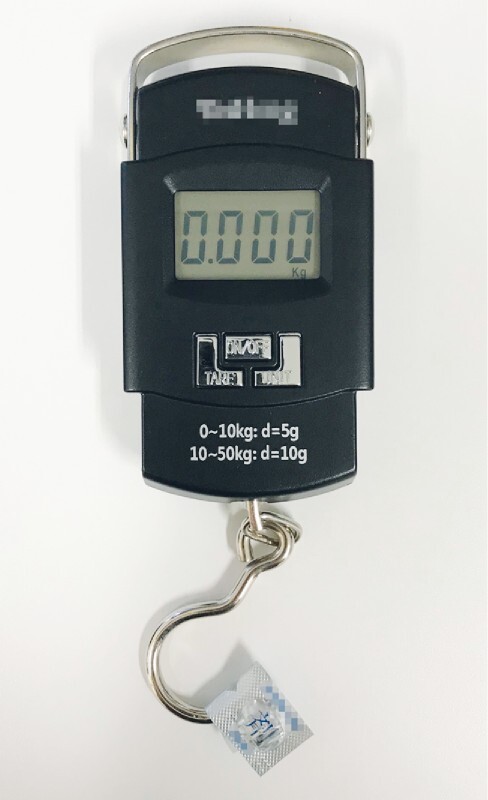
A PTP sheet was attached to the tip of the spring scale to evaluate the gripping force of various devices.

**Fig. 3:**
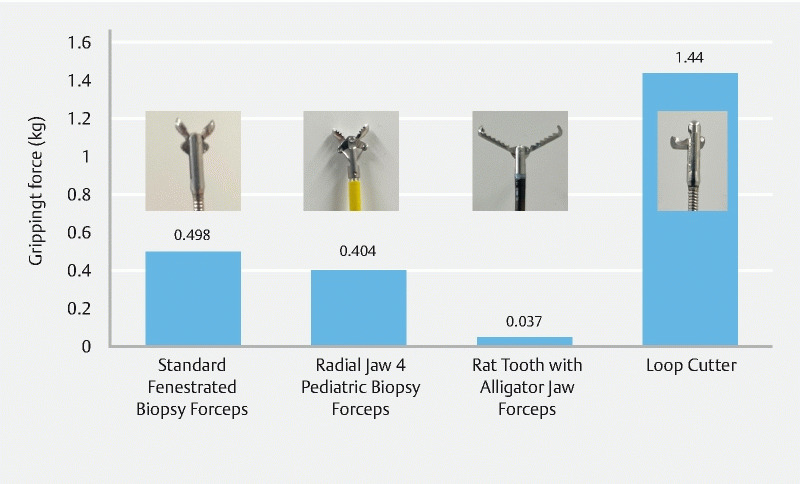
Comparison of the gripping force of various devices.

A 70-year-old woman presented to our hospital complaining of a sore throat after accidentally ingesting PTP sheets. A computed tomography (CT) scan showed the PTP location in the esophagus ( Fig. 4 ). The second case was a 63-year-old man with dementia. PTP was found in the stomach along with a large amount of food residue. The PTP sheets were successfully and safely removed endoscopically in both cases by grasping the PTP sheet using a Loop Cutter ( Fig. 5 , Video 1 ).

**Fig. 4:**
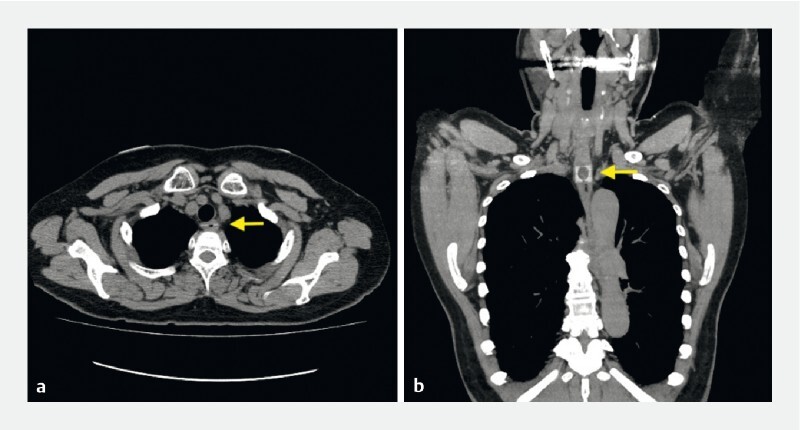
A 70-year-old woman presented to our hospital complaining of a sore throat after accidentally ingesting PTP sheets. **a, b** A computed tomography scan showed the PTP sheets located in the esophagus.

**Fig. 5 a:**
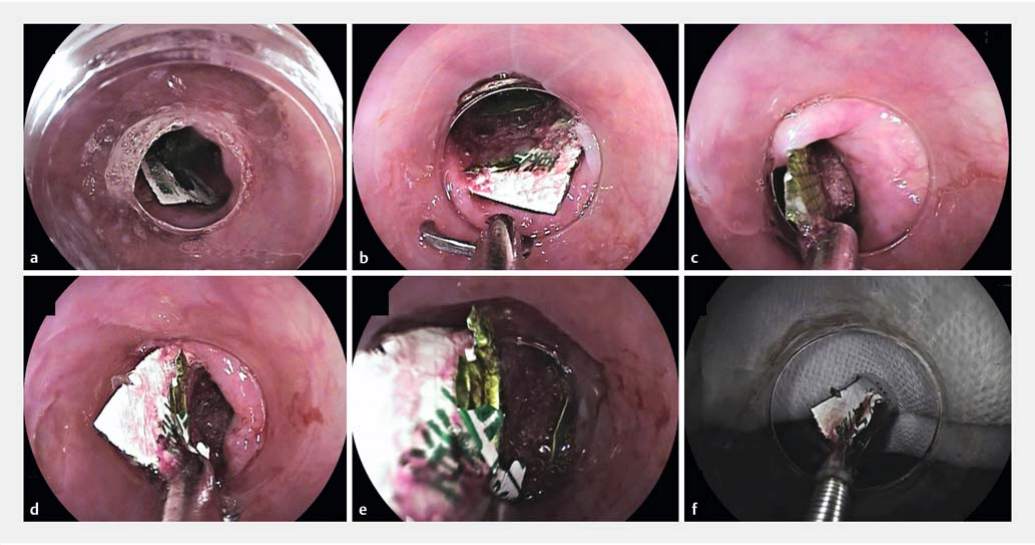
The ingested PTP sheet was located in the upper esophagus. **b–e** We used the Loop Cutter. Its teeth can penetrate the PTP plastic sheet, securing a firm grip. Because the gripping force is strong, the PTP can be easily removed. **f** The PTP sheet was detached easily from the Loop Cutter.

**Video 1** A Loop Cutter is an ideal gripper for endoscopic removal of press-through packages.


Endoscopy_UCTN_Code_TTT_1AO_2AL

## Notes

Endoscopy E-Videos

https://eref.thieme.de/e-videos
*E-Videos* is an open access online section of the journal *Endoscopy* , reporting on interesting cases and new techniques in gastroenterological endoscopy. All papers include a high-quality video and are published with a Creative Commons CC-BY license. Endoscopy E-Videos qualify for HINARI discounts and waivers and eligibility is automatically checked during the submission process. We grant 100% waivers to articles whose corresponding authors are based in Group A countries and 50% waivers to those who are based in Group B countries as classified by Research4Life (see: https://www.research4life.org/access/eligibility/ ). This section has its own submission website at https://mc.manuscriptcentral.com/e-videos
